# Fungal Community Composition Varies Across Floral and Extrafloral Nectaries

**DOI:** 10.1002/jobm.70193

**Published:** 2026-08-05

**Authors:** Jefferson Brendon Almeida dos Reis, Sofia Coradini Schirmer, Helson Mario Martins do Vale

**Affiliations:** ^1^ Department of Phytopathology Postgraduate Program in Microbiology Biology, Institute of Biological Sciences, University of Brasília (UnB) Brasília Brazil; ^2^ Department of Zoology, Postgraduate Program in Ecology, Institute of Biological Sciences University of Brasília (UnB) Brasília Brazil; ^3^ Department of Phytopathology, Institute of Biological Sciences University of Brasília (UnB) Brasília Brazil

**Keywords:** *Cladosporium*, *Hymenaea stigonocarpa*, *Maprounea brasiliensis*, metabarcoding, phyllosphere, *Starmerella*, yeast

## Abstract

Floral (FNs) and extrafloral nectaries (EFNs) are key plant microhabitats that mediate interactions among plants, insects, and microorganisms, yet their associated fungal communities remain poorly characterized, especially in tropical ecosystems. Here, we investigated the diversity, taxonomic composition, and functional structure of fungal communities across distinct plant microhabitats of two woody Cerrado species, *Maprounea brasiliensis* and *Hymenaea stigonocarpa*, using an environmental DNA metabarcoding approach. In *M. brasiliensis*, fungal communities from floral and extrafloral nectaries were compared, whereas in *H. stigonocarpa*, extrafloral nectaries were contrasted with adjacent phyllosphere and endosphere compartments. Across all microhabitats, communities were dominated by Ascomycota, with *Cladosporium* as the most abundant genus. Yeast genera such as *Hannaella, Starmerella*, and *Aureobasidium*, although less abundant than filamentous fungi, were also consistently detected as part of the core mycobiota across both plant species and compartments. In *M. brasiliensis*, EFNs exhibited higher richness, diversity, and evenness than FNs, and beta diversity analyses revealed clear compositional differentiation between these nectary types. In contrast, no significant differences in alpha or beta diversity were detected between EFNs and phyllosphere/endosphere compartments of *H. stigonocarpa*. Functional profiles were dominated by plant pathogenic and saprotrophic fungi in all microhabitats, with filamentous forms prevailing over yeasts. However, yeast‐like growth forms were relatively more abundant in the EFNs of *M. brasiliensis*. These results show that floral and extrafloral nectaries act as ecologically distinct microhabitats that contribute to fungal community structuring in Cerrado plants, with implications for plant–insect–microbe interactions in a highly diverse and understudied biome.

**Abbreviations**
ANOSIM: analysis of similarities
ASV: amplicon sequence variant
CTAB: cetyltrimethylammonium bromide
DNA: deoxyribonucleic acid
eDNA: environmental DNA
EDTA: ethylenediaminetetraacetic acid
EFN/EFNs: extrafloral nectary/extrafloral nectaries
FN/FNs: floral nectary/floral nectaries
ITS: internal transcribed spacer
NMDS: non‐metric multidimensional scaling
PERMANOVA: permutational multivariate analysis of variance
PVP: polyvinylpyrrolidone
RECOR/IBGE: ecological reserve of the brazilian institute of geography and statistics
TE: tris–EDTA buffer
UV: ultraviolet

## 1 Introduction

Floral and extrafloral nectaries are secretory structures widely distributed across different plant lineages, having evolved multiple times independently and displaying remarkable morphological and functional diversity [1, 2, 3, 4]. Floral nectaries (FNs) are directly involved in reproduction, mediating interactions with pollinators through the provision of nectar rich in sugars and amino acids [5, 6, 7]. Extrafloral nectaries (EFNs) serve important ecological functions, such as recruiting ants and other arthropod defenders that can reduce herbivory [8, 9, 10, 11, 12]. In addition to these specialized organs, the phyllosphere (the aerial leaf surface) and the endosphere (the internal tissue environment) represent important microbial habitats [13, 14, 15, 16, 17]. Collectively, FNs, EFNs, the phyllosphere, and the endosphere constitute key ecological interfaces among plants, animals, bacteria, and fungi, shaping both plant ecology and the dynamics of associated communities.

Based on the recognition of FNs, EFNs, the phyllosphere, and the endosphere as key ecological compartments, their associated microbial communities have been increasingly studied in recent decades. In the case of FNs, recent research has focused on characterizing their resident microbiota to better understand interactions among plants, microorganisms, and pollinators [18, 19, 20, 21]. Those studies have primarily examined yeasts and bacteria, assessing their prevalence and abundance, as well as their influence on the physicochemical properties of nectar and, consequently, on pollinator visitation rates [22, 23, 24, 25]. For EFNs, although less studied, there is evidence that they also function as microbial microhabitats [26, 27, 28], potentially modulating plant–defender interactions. The phyllosphere and endosphere, in turn, are recognized as dynamic environments hosting highly diverse microbial communities that are sensitive to both biotic and abiotic factors [16, 17, 29, 30, 31, 32, 33]. Nevertheless, little is known about how these four compartments, FNs, EFNs, the phyllosphere, and the endosphere, compare in terms of fungal composition, particularly in species native to tropical ecosystems such as the Cerrado.

The Brazilian Cerrado is recognized as a global biodiversity hotspot, characterized by high plant richness [34, 35, 36, 37], marked seasonality [38], and nutrient‐poor soils [39]. Floral and extrafloral nectaries of Cerrado plants have been predominantly investigated from a morphoanatomical perspective [40, 41] and for their role in mediating interactions with arthropods [42, 43, 44, 45, 46], while their associated microbial communities remain poorly studied. Although significant progress has been made in understanding the mycobiota of plant species in natural ecosystems [29], to the best of our knowledge, studies addressing fungal communities in FNs and EFNs have generally examined these structures in isolation, without assessing which microorganisms are unique to each niche. Moreover, no reports have investigated these microhabitats through metabarcoding approaches in parallel with adjacent compartments, such as the leaf endosphere and phyllosphere. This gap is particularly relevant because fungi can influence key processes, from modifying the physicochemical properties of nectar to regulating the behavior of pollinators, ants, and other associated organisms [18, 19, 20, 21]. Thus, investigating how fungal communities are structured and distributed within these compartments, within a single plant or among distinct species, may reveal underexplored ecological mechanisms and clarify their role in nectary‐mediated interactions.

Considering these knowledge gaps, we formulated central hypotheses regarding the structure and composition of fungal communities associated with nectaries. For *Maprounea brasiliensis* A. St.‐Hil. (Euphorbiaceae), we hypothesized that: (a1) fungal communities associated with floral and extrafloral nectaries differ in taxonomic composition and community structure; (a2) floral nectaries exhibit higher taxon richness due to recurrent visitation by pollinators belonging to distinct taxonomic groups; (a3) fungal functional guild composition differs between floral and extrafloral nectaries; (a4) filamentous growth forms are more frequent in floral nectaries than in extrafloral nectaries; and (a5) specific fungal taxa are associated with each nectariferous microhabitat. These hypotheses were tested through the characterization of the mycobiota associated with floral (inflorescences) and extrafloral nectaries of this species. For *Hymenaea stigonocarpa* Mart. ex Hayne (Fabaceae), we adopted a descriptive approach, focusing on the taxonomic profiling of fungal communities associated with extrafloral nectaries, phyllosphere/endosphere. Given the exploratory nature of this dataset and the limitations in sampling, no a priori hypotheses were formulated for this species, and the results should be interpreted as a preliminary characterization of fungal communities across these plant compartments.

## 2 Materials and Methods

### 2.1 Collection and Sampling

We collected individuals of *M. brasiliensis* and *H. stigonocarpa* on August 27, 2025, during the peak of the dry season, in firebreaks within a Cerrado *sensu stricto* area at the Ecological Reserve of the Brazilian Institute of Geography and Statistics (RECOR/IBGE) (15°55′ S, 47°51′ W), approximately 35 km from Brasília, Federal District, Brazil. The regional climate is tropical seasonal (Aw), characterized by two well‐defined seasons: dry, from April to September, and rainy, from October to March [38]. The local vegetation corresponds to Cerrado *sensu stricto*, consisting of a matrix of grasses, shrubs, and woody tree species typical of the biome.

**Figure 1:**
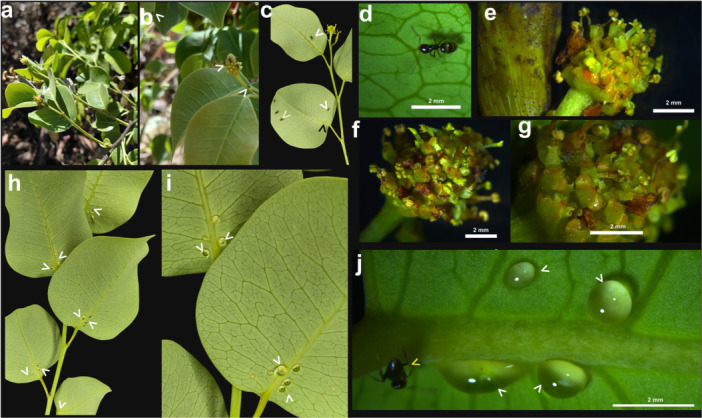
Morphology of *Maprounea brasiliensis* A. St.‐Hil. (Euphorbiaceae) and details of its floral and extrafloral nectaries. (a) Leaves and (b) inflorescence of *M. brasiliensis* in natural field conditions; the arrow in (b) indicates the inflorescence. (c, d) Ants foraging on the leaf surface. (e–g) Details of the inflorescences. (h, i) Exposed extrafloral nectaries on the abaxial surface and at the leaf base, shown in active secretion with accumulated nectar (white arrows). (j) Extrafloral nectaries producing nectar (white arrows), with an ant foraging nearby (yellow arrow). The images were acquired using the Microbes, smile for the picture (MSFP) protocol [47].

**Figure 2:**
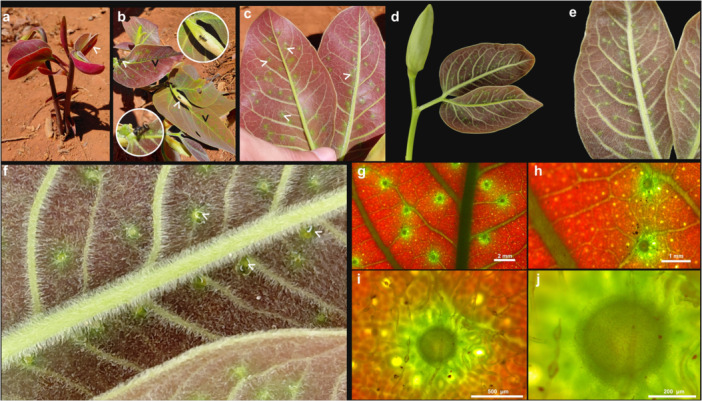
Morphology of *Hymenaea stigonocarpa* Mart. ex Hayne (Fabaceae) and details of its extrafloral nectaries. (a) Individual of *H. stigonocarpa* under natural field conditions; the white arrow indicates an ant foraging among the leaves. (b) Different ant species foraging on the leaves (white arrows), with visible nectaries indicated by black arrows. (c) Abaxial leaf surface showing numerous extrafloral nectaries (white arrows). (d, e) Arrangement and distribution of extrafloral nectaries on the abaxial surface. In (e), nectaries in active secretion with accumulated nectar are visible (white arrows). (g–j) Detailed view of floral nectaries. The images were acquired using the Microbes, smile for the picture (MSFP) protocol [47].

We selected plant species based on their widespread distribution in the Cerrado and the presence of easily distinguishable EFNs on the abaxial leaf surface. Both are woody species endemic to the biome, with clearly visible nectariferous structures, which facilitated sampling. To minimize pseudoreplication, individuals were selected at least 30 m apart. We sampled *M. brasiliensis* during both vegetative and reproductive phenophases, when plants simultaneously exhibited inflorescences and active EFNs on newly expanded leaves (Figure 1). We collected five individuals ranging from 40 to 50 cm in height. From each plant, we obtained 30–60 newly expanded leaves with active EFNs and 15–30 inflorescences. For *H. stigonocarpa*, we sampled three individuals ranging from 15 to 35 cm in height. Each plant had 4–10 leaves bearing active EFNs (Figure 2). All collected material (leaves and inflorescences) was placed in sterile plastic bags, labeled the samples, and stored in insulated boxes at a controlled temperature (~18°C). Samples were then transported to the laboratory and processed within 24 h of collection.

### 2.2 Environmental DNA Extraction and Sequencing

**Figure 3:**
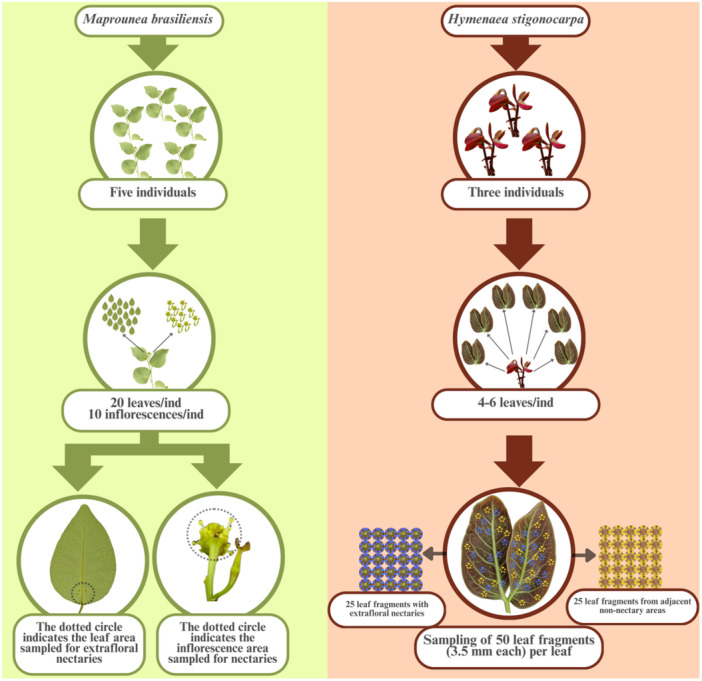
Sampling design for fungal community analysis in *Maprounea brasiliensis* and *Hymenaea stigonocarpa*. For *M. brasiliensis*, leaves and inflorescences were sampled at nectary sites (dotted circles). For *H. stigonocarpa*, leaf fragments with and without extrafloral nectaries were collected.

The type of material used for environmental DNA (eDNA) extraction varied depending on the species and tissue type. For *M. brasiliensis*, eDNA was obtained from leaves and inflorescences containing nectaries (Figure 3). From each individual (*n* = 5), 20 leaves were collected, each bearing at least 2 pairs of nectaries (Figure 3). Leaf regions containing nectaries were carefully excised using a sterile scalpel, transferred to 50 mL Falcon tubes containing 3 mL of TE High buffer (10 mM Tris‐HCl, pH 8.0; 1 mM EDTA), and immediately stored at –80°C until extraction. Additionally, 10 inflorescences per individual were collected, sectioned at the peduncle, placed in TE High buffer, and stored under the same conditions. For *H. stigonocarpa*, 4–6 leaves per individual (*n* = 3) were collected (Figure 3). From each leaf, 3.5 mm circular fragments were obtained, yielding a total of 25 fragments containing nectaries and 25 fragments from adjacent regions without extrafloral nectaries. All fragments were stored in TE High buffer at –80°C until processing.

We extracted DNA following the protocol of dos Reis et al. [47], with modifications. Mortars and pestles were pre‐cooled in liquid nitrogen, and plant material was added to the chilled crucibles and pulverized until a fine powder was obtained. Approximately 250 mg of ground tissue was transferred to 2 mL microtubes containing 1 mL of preheated CTAB buffer (7%–10% CTAB, 100 mM Tris‐HCl pH 8.0, 20 mM EDTA, 1.4 M NaCl) supplemented with 75 mg of PVP‐40. Samples were incubated at 65°C with shaking at 900 rpm for 15 min, with brief vortexing every 3 min. Tubes were centrifuged at 16,000 rpm for 10 min at 10°C, and 900 µL of the supernatant was transferred to new microtubes. Subsequently, 150 µL of buffer and 600 µL of chloroform:isoamyl alcohol (24:1) was added, gently mixed for 10 min, and centrifuged again under the same conditions. The aqueous phase (~900 µL) was recovered and subjected to a second extraction step to ensure the complete removal of the interface. Approximately 800 µL of the final aqueous phase was transferred to microtubes containing an equal volume of cold isopropanol, mixed by inversion, and stored at –20°C for 2 h. Samples were then centrifuged at 16,000 rpm at 4°C to precipitate DNA. The supernatant was discarded, and the pellet was washed sequentially with 70% ethanol (400 µL) and absolute ethanol (700 µL), with centrifugation for 5 min at 4°C between washes. After drying at room temperature for 1 h, DNA was resuspended in 100 µL of TE buffer. DNA integrity and quality were assessed on a 1% (w/v) agarose gel stained with GelRed and visualized under UV light. The ITS1 region was selected as the target genetic marker and amplified using the primer pair ITS1‐1F‐F (5′–CTTGGTCATGGGAAGTAA–3′) and ITS1‐1F‐R (5′–GCTGCGTCTCTCGTGC–3′). Sequencing was performed using the Illumina MiSeq. 2500 platform (Illumina, San Diego, CA, USA).

### 2.3 Downstream Bioinformatics Analysis for ASVs Generation

Sequencing data were initially inspected to assess the overall quality of the generated reads, as reported by the sequencing service provider (Material S1—Table S1). Read quality and adapter content were assessed using FastQC (v0.11.9) [48], and sequences were processed using DADA2 (v1.32.0) [49] in R (v4.4.0). Adapters were removed using Cutadapt (v4.8) [50] via its integrated plug‐in. Demultiplexed, adapter‐free reads were then processed following the DADA2 ITS workflow with minor modifications (https://benjjneb.github.io/dada2/ITS_workflow.html/access in January 2026). Quality filtering was performed using the *filterAndTrim* function with the parameters ith *maxEE* = *c(2,5)*, *truncQ* = *5*, and *minLen* = *100*. Reads were truncated at the first base with a Phred score below the truncQ threshold, and reads with expected errors equal to or greater than the maxEE threshold were discarded. Amplicon sequence variants (ASVs) were inferred using the DADA2 algorithm, forward and reverse reads were merged, and chimeric sequences were removed. Taxonomic assignment was performed using the UNITE general [51] FASTA release for eukaryotes (*sh_general_release_dynamic_all_19.02.2025.fasta*) [52]. Finally, a post‐processing table was generated, and the proportions of input and retained sequences were calculated to evaluate filtering and processing efficiency (Table S2).

### 2.4 Analysis of Ecological Data

We conducted ecological analyses in RStudio (v.4.4.0). ASV tables, taxonomic assignments, and environmental metadata were integrated into a *phyloseq* object using the *phyloseq* package (v.1.52.0). ASVs not assigned to the kingdom Fungi were removed, and the dataset was subsequently converted to the microeco format using the *file2meco* package (v.0.80).

We evaluated whether variation in sequencing depth could bias downstream analyses and whether rarefaction was necessary [53, 54, 55, 56] by comparing the number of non‐chimeric reads per sample after denoising. Rarefaction curves for each sample were generated using the *vegan* package (v.2.6‐8) [57]. When curves did not reach a plateau, a resampling procedure was applied to reduce the effects of uneven sequencing depth [58], using scaling‐based rarefaction with stratified subsampling [55, 56, 59] implemented via the “*rarefy_sample*” function and the rarefy method in *microeco* (v.1.9.1). Sampling adequacy and coverage of fungal diversity across microhabitats were further assessed using rarefaction and extrapolation curves generated with *iNEXT* (v.3.0.1) [60].

Relative abundances at different taxonomic levels were calculated using the *trans_abund* class (*microeco* v.1.9.1). Alpha diversity was estimated using observed richness, Shannon and Simpson indices, and Pielou's evenness via the *trans_alpha* class [47, 61, 62]. Alpha diversity metrics were compared among plant microhabitats to assess differences in fungal community structure. Normality was evaluated for each metric and microhabitat using the Shapiro–Wilk test. Given the small sample sizes and the lack of consistent normality, comparisons were conducted using the non‐parametric Wilcoxon rank‐sum test. To account for multiple testing, p‐values were adjusted using the Benjamini–Hochberg procedure. Effect sizes were estimated using Cliff's delta to quantify the magnitude of differences between microhabitats.

Beta diversity was assessed using Bray–Curtis and Jaccard distance metrics and visualized by non‐metric multidimensional scaling (NMDS) ordinations using the *trans_beta* class. Homogeneity of multivariate dispersion was evaluated using β‐dispersion with 9,999 permutations [63]. Differences in community composition among microhabitats were tested using ANOSIM and PERMANOVA with 9,999 permutations [64, 65]. Beta diversity was further partitioned into turnover and nestedness components using the *betapart* package (v.1.6) to infer the ecological processes underlying community differentiation among microhabitats [66].

Indicator species analysis was performed to identify ASVs associated with distinct microhabitats using the *indicspecies* package (v.1.8.0). ASVs occurring in fewer than two samples were excluded prior to analysis. Indicator ASVs were identified using multilevel pattern analysis implemented in the “*multipatt*” function with the IndVal.g index, which integrates specificity and fidelity. Statistical significance was assessed using 999 permutations, and only ASVs with *p* ≤ 0.05 were considered significant.

To identify stable and recurrent fungal taxa across samples and microhabitats, a core mycobiote analysis was conducted using ASVs data processed in *phyloseq*. A pseudocount (1 × 10^−6^) was added prior to transformation into relative abundances using “*microbiome::transform”* (*microbiome* v.1.26.0) to avoid zero inflation. Taxonomic assignments were refined using “*microbiome::add_besthit.*” The core community was defined as ASVs present in at least 70% of samples with a minimum relative abundance of 0.01%. Core community composition was visualized using line plots and heatmaps. This analysis was performed at both the ASV and genus levels, and unclassified genera (“Unknown”) were removed to ensure that observed patterns reflected only taxonomically resolved groups.

Functional profiling of the fungal community was performed using a microtable object implemented in the *microeco* package. Initially, the dataset was organized and standardized, and fungal ecological functions were inferred using the FungalTraits database, which assigns functional attributes based on taxonomic information [67]. Functional traits were calculated using the *trans_func* class, generating tables of raw functional abundance and relative abundance (%). The resulting data were subsequently transformed into a long format to facilitate statistical analyses and graphical visualization. Functional categories were grouped according to primary lifestyle, secondary lifestyle, and growth form. Differences in the relative abundance of functional categories were assessed using the non‐parametric Wilcoxon rank‐sum test, which is appropriate for data that do not meet normality assumptions.

## 3 Results

### 3.1 Sequencing and Rarefaction

Across all libraries, more than 96% of the reads exhibited Q‐scores ≥20, and over 91% showed Q‐scores ≥30 (Table S1), indicating high sequencing quality. After paired‐end merging, quality filtering, and chimera removal, a total of 2,334,956 high‐quality sequences were retained (Table S2). Rarefaction analyses showed that all samples from *M. brasiliensis* reached clear plateaus, indicating sufficient sequencing depth to capture most of the fungal diversity (Figure S1a). In contrast, not all samples from *H. stigonocarpa* reached a plateau, suggesting incomplete coverage of fungal diversity in this host (Figure S1b). Additionally, one phyllosphere/endosphere replicate of *H. stigonocarpa* was lost during shipment, resulting in a reduced number of biological replicates. Rarefaction and extrapolation curves based on Hill number q0 for extrafloral and floral nectaries of *M. brasiliensis* revealed well‐defined plateaus, with extrapolation indicating only marginal gains in estimated richness (Figure S2a). For *H. stigonocarpa*, extrafloral nectaries exhibited high ASV richness with curves approaching saturation, whereas the adjacent phyllosphere/endosphere displayed lower richness and less stabilized curves, indicating that sequencing depth may have been insufficient to fully characterize the fungal diversity in this compartment (Figure S2b).

### 3.2 Relative Abundance and Taxonomic Composition of Mycobiota

**Figure 4:**
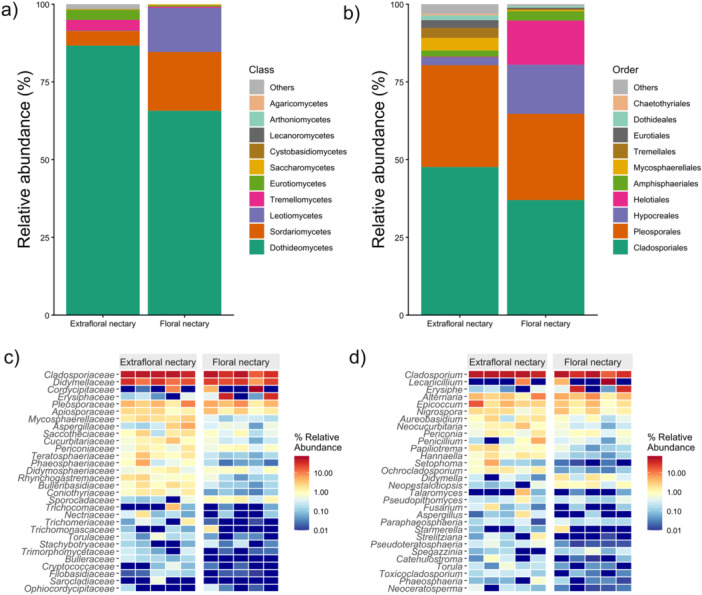
Taxonomic composition of fungal communities associated with extrafloral and floral nectaries of *Maprounea brasiliensis*. (a) Relative abundance of fungal classes and (b) fungal orders, highlighting shifts in dominant taxonomic groups between microhabitats. (c) Heatmap showing the relative abundance of the most abundant fungal families and (d) genera across individual samples from extrafloral and floral nectaries. Color gradients represent relative abundance (log10‐transformed scale), with red color indicating higher abundances.

The mycobiota associated with FNs and EFNs of *M. brasiliensis* was dominated by Ascomycota, accounting for 95.9% of sequences in EFNs and 99.6% in FNs. EFN‐associated communities were strongly dominated by Dothideomycetes (86.6%), with Sordariomycetes occurring at much lower abundance (4.8%) (Figure 4a). In contrast, FNs showed a more heterogeneous composition, with Dothideomycetes (65.7%), Sordariomycetes (18.9%), and Leotiomycetes (14.2%). At the order level, Cladosporiales and Pleosporales predominated in EFNs (47.6% and 32.8%, respectively) (Figure 4b). FNs displayed a similar pattern, although with lower proportions of Cladosporiales (37.0%) and Pleosporales (27.8%), and a higher contribution of Hypocreales (15.8%). At the family level, EFNs were dominated by few taxa, mainly Cladosporiaceae (47.6%) and Didymellaceae (21.4%), while the remaining families (< 2%) accounted for 29.2% (Figure 4c). In FNs, Cladosporiaceae (37.0%), Didymellaceae (21.1%), and Cordycipitaceae (15.5%) were the most abundant, with taxa below 5% collectively representing 26.4%. At the genus level, *Cladosporium* was the most abundant in both nectary types (47.4% in EFNs; 36.9% in FNs), while *Lecanicillium* (15.5%) and *Erysiphe* (14.2%) also contributed substantially in FNs (Figure 4d).

**Figure 5:**
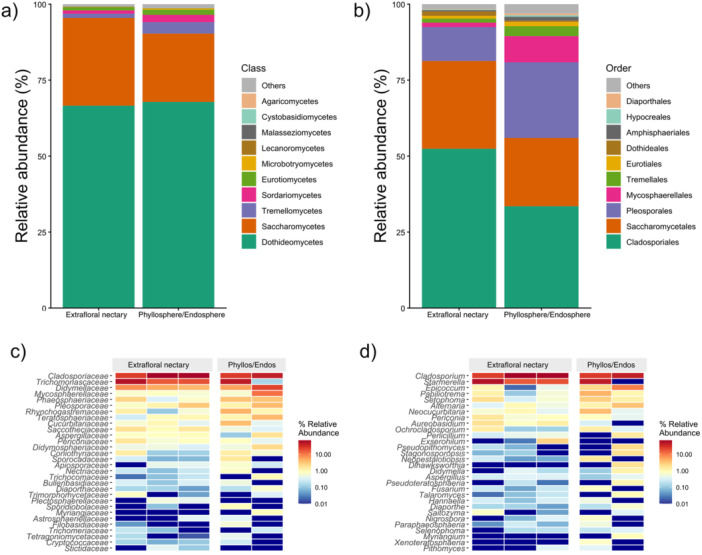
Taxonomic composition of fungal communities associated with extrafloral nectaries and adjacent phyllosphere and endosphere of *Hymenaea stigonocarpa*. (a) Relative abundance of fungal classes and (b) fungal orders, highlighting shifts in dominant taxonomic groups between microhabitats. (c) Heatmap showing the relative abundance of the most abundant fungal families and (d) genera across individual samples from extrafloral nectaries and adjacent phyllosphere and endosphere. Color gradients represent relative abundance (log10‐transformed scale), with red color indicating higher relative abundances.

The mycobiota associated with EFNs and the adjacent phyllosphere/endosphere of *Hymenaea stigonocarpa* was dominated by Ascomycota, accounting for 98.4% and 95.4% of the sequences, respectively. Dothideomycetes predominated in both microhabitats (66.6% in EFNs; 67.8% in the phyllosphere/endosphere), followed by Saccharomycetes (28.9% and 22.5%, respectively) (Figure 5a). At the order level, Cladosporiales, Saccharomycetales, and Pleosporales were the most abundant in EFNs (52.4%, 28.9%, and 11.2%, respectively), while the phyllosphere/endosphere showed a similar composition, with Cladosporiales (33.5%), Pleosporales (24.9%), and Saccharomycetales (22.5%) predominating. Mycosphaerellales (8.6%) and Tremellales (3.3%) also exceeded 2% relative abundance in the phyllosphere/endosphere (Figure 5b). At the family level, Cladosporiaceae dominated both microhabitats (52.4% in EFNs; 33.5% in the phyllosphere/endosphere), followed by Trichomonascaceae (28.8%) and Didymellaceae (5.4%) in EFNs, and by Trichomonascaceae (33.5%) and Didymellaceae (13.7%) in the phyllosphere/endosphere (Figure 5c). At the genus level, *Cladosporium* was the most abundant genus in both microhabitats, while *Starmerella* was the second most abundant in EFNs (28.8%) (Figure 5d).

### 3.3 Alpha Diversity

Fungal communities associated with nectaries and leaf compartments of the two plant species analyzed showed variation across the evaluated alpha diversity indices (Figure S3). In *M. brasiliensis*, EFNs exhibited higher mean values of observed richness (237 ± 65.7) compared to FNs (158 ± 41.1). For this species, Pielou (0.549 ± 0.0488 vs. 0.408 ± 0.0881), Shannon (2.97 ± 0.219 vs. 2.07 ± 0.508), and Simpson (0.858 ± 0.0289 vs. 0.738 ± 0.163) indices also showed higher mean values in EFNs relative to FNs. Statistical comparisons indicated significant differences between microhabitats for Pielou (adjusted *p* = 0.0318), Shannon (adjusted *p* = 0.0318), and Simpson (adjusted *p* = 0.0423), whereas observed richness did not differ significantly (adjusted *p* = 0.095). Effect size estimates based on Cliff's delta indicated complete separation between microhabitats for all evaluated metrics (*δ* = 1 or *δ* = −1). In *H. stigonocarpa*, observed richness was higher in EFNs (148 ± 21.4) than in the phyllosphere/endosphere (62 ± 7.07). In contrast, Pielou, Shannon, and Simpson indices were higher in the phyllosphere/endosphere. However, none of the alpha diversity metrics differed significantly between microhabitats (adjusted *p* = 0.2).

### 3.4 Beta Diversity

**Figure 6:**
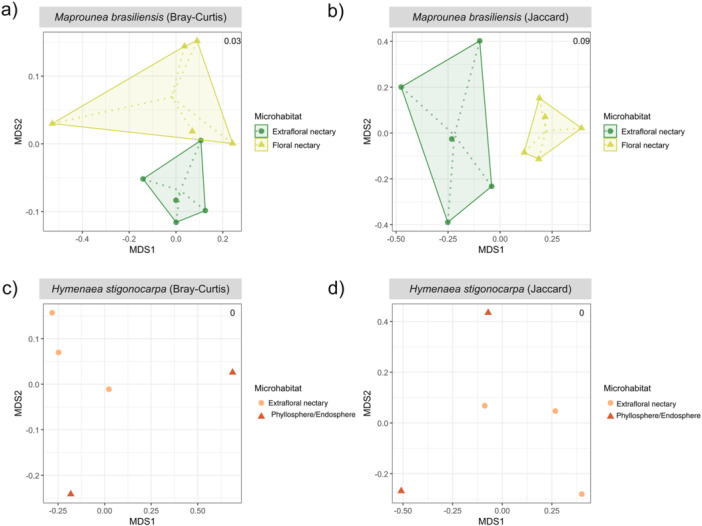
Non‐metric multidimensional scaling (NMDS) ordinations based on Bray–Curtis (a, c) and Jaccard (b, d) dissimilarities showing fungal community composition associated with different plant microhabitats of two native species from the Brazilian Cerrado. (a, b) represent communities from extrafloral and floral nectaries of *M. brasiliensis*. (c, d) represent communities from extrafloral nectaries and the phyllosphere/endosphere of *H. stigonocarpa*. Symbols indicate microhabitats.

We found that the beta diversity of communities associated with *M. brasiliensis* differed between extrafloral and floral nectaries, indicating that these microhabitats function as ecologically distinct niches. Ordination analyses based on Bray–Curtis dissimilarity showed a clear tendency for samples to cluster according to microhabitat, with an excellent representation of community structure (Figure 6a,b; NMDS stress = 0.034). Abundance‐based analyses revealed consistent differences between extrafloral and floral nectaries, as supported by a significant effect of microhabitat in PERMANOVA (*R* ^2^ = 0.189, *p* = 0.03) and ANOSIM (*R* = 0.256, *p* = 0.03), with no differences in dispersion between groups (betadisper, *p* = 0.536). In contrast, presence–absence‐based analyses using the Jaccard index revealed stronger compositional differentiation between nectary types (PERMANOVA: *R* ^2^ = 0.178, *p* = 0.007; ANOSIM: *R* = 0.56, *p* = 0.006), but also significantly different dispersions between microhabitats (betadisper, *p* = 0.001). In *H. stigonocarpa*, beta diversity analyses revealed no significant differences in fungal community composition between extrafloral nectaries and the phyllosphere/endosphere, regardless of whether abundance‐based (Bray–Curtis: PERMANOVA *R* ^2^ = 0.35; ANOSIM *R* = 0.5; *p* > 0.1) or presence–absence metrics were used (Jaccard: PERMANOVA *R* ^2^ = 0.28; ANOSIM *R* = 0.5; *p* > 0.1). Community dispersion also did not differ between microhabitats (*p* > 0.1) (Figure 6c,d).

The Sørensen beta diversity for *M. brasiliensis* indicated moderate compositional dissimilarity between the microbial communities associated with floral and extrafloral nectaries (βSOR = 0.44). Community differentiation was predominantly driven by species turnover (βSIM = 0.36), whereas nestedness contributed less to overall beta diversity (βSNE = 0.08). Pairwise comparisons revealed lower dissimilarity among samples within the same nectary type and higher dissimilarity between nectary types, supporting the existence of distinct nectar‐associated communities (Figure S4a). For *H. stigonocarpa*, Sørensen beta diversity also suggested moderate dissimilarity among fungal communities associated with extrafloral nectaries, the phyllosphere, and the endosphere (βSOR = 0.46). Community differentiation was mainly driven by species turnover (βSIM = 0.36), while nestedness played a minor role (βSNE = 0.10), indicating that plant compartments harbor distinct communities rather than hierarchical subsets of a common species pool (Figure S4b).

### 3.5 Indicator Species Analysis

**Figure 7:**
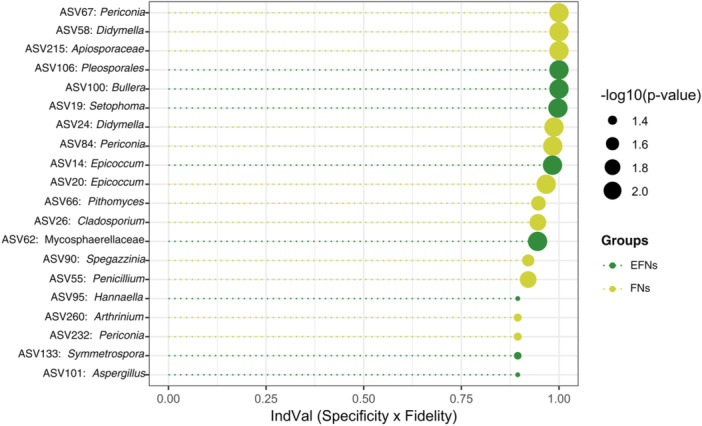
Indicator species analysis (IndVal) showing ASVs significantly associated with floral (FNs) and extrafloral nectaries (EFNs) of *Maprounea brasiliensis*. Points represent indicator ASVs, with color indicating the nectary type (green = EFNs; yellow = FNs) and point size proportional to statistical significance (−log10 *p*‐value). Values on the *x*‐axis represent IndVal scores, with higher values indicating stronger association with a given nectary type.

Indicator species analysis (IndVal) identified 23 ASVs significantly associated with nectary types (Figure 7; *p* ≤ 0.05), supporting microbial differentiation between floral and extrafloral nectaries of *M. brasiliensis*. Extrafloral nectaries were associated with eight indicator ASVs showing high specificity and fidelity, including representatives of both Ascomycota and Basidiomycota. Floral nectaries harbored 12 indicator ASVs with strong statistical support. No significant indicator ASVs were detected for the microhabitats of *H. stigonocarpa*, likely due to the low sample size and incomplete sampling coverage indicated by the rarefaction curves.

### 3.6 Core of Mycobiota

A recurrence analysis based on relative abundance and prevalence identified a consistent core of fungal taxa shared between FNs and EFNs of *M. brasiliensis* (Figure S5a–c). Using a minimum detection threshold of 0.01% relative abundance and prevalence ≥70% of samples, 38 ASVs were detected, representing the most stable fraction of the nectary‐associated mycobiota. These ASVs were predominantly affiliated with Ascomycota, mainly within Dothideomycetes (genera = *Cladosporium, Alternaria, Epicoccum, Nigrospora, Periconia*, and *Aureobasidium*). Basidiomycetous yeasts, including *Papiliotrema and Hannaella*, were also detected. For *H. stigonocarpa*, recurrence analysis revealed a stable fungal core associated with EFNs and the adjacent phyllosphere and endosphere (Figure S5b–d). Core ASVs were mainly affiliated with Ascomycota, with dominance of Dothideomycetes and Saccharomycetes.

### 3.7 Guilds Function and Trophic Modes

**Figure 8:**
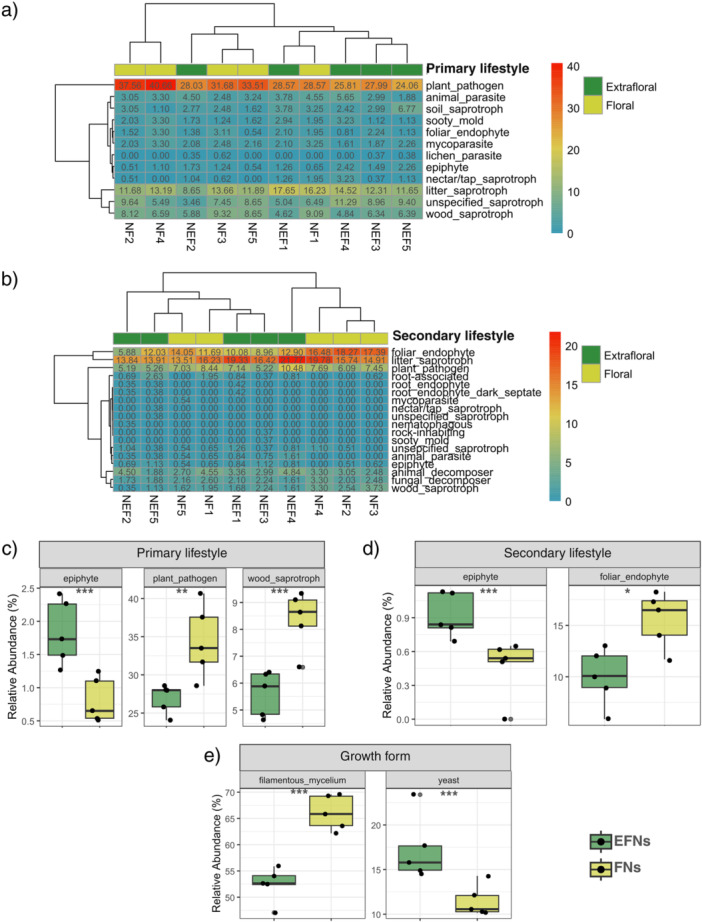
Functional structure of fungal communities associated with extrafloral (EFNs) and floral nectaries (FNs) of *Maprounea brasiliensis*. (a) Heatmap and hierarchical clustering of primary lifestyles based on relative abundance (%). (b) Heatmap and hierarchical clustering of secondary lifestyles based on relative abundance (%). (c) Boxplots showing significant differences in primary lifestyles between EFNs and FNs. (d) Boxplots showing significant differences in secondary lifestyles between EFNs and FNs. (e) Boxplots comparing growth forms (filamentous mycelium and yeast) between nectary types. Statistical significance was assessed using the Wilcoxon rank‐sum test (*p < 0.05; **p < 0.01; ***p < 0.001).

The fungal community associated with EFNs and FNs of *M. brasiliensis* exhibited high functional diversity, comprising 12 primary lifestyles and 18 secondary functional guilds (Figure 8a,b). The plant pathogen lifestyle was dominant in both microhabitats, followed by litter saprotrophs, unspecified saprotrophs, and wood saprotrophs. Significant differences between EFNs and FNs were detected for the primary lifestyles epiphytic, plant pathogenic, and wood saprotrophic (*p* < 0.05) (Figure 8c). The epiphytic lifestyle was more abundant in EFNs, whereas plant pathogens and wood saprotrophs showed higher relative abundance in FNs. Regarding secondary lifestyles, fungal communities in both EFNs and FNs were dominated by litter saprotrophs, followed by foliar endophytes and plant pathogens. Among these categories, epiphytic and foliar endophytic lifestyles differed significantly between microhabitats (*p* < 0.05) (Figure 8d). Epiphytic fungi were more abundant in EFNs, while foliar endophytes predominated in FNs. In terms of growth form, three modes were identified in EFNs and FNs of *M. brasiliensis*: filamentous, yeast, and dimorphic yeast. The filamentous growth form was dominant in FNs, accounting for more than 60% of the community in all samples, and was significantly higher than in EFNs (Figure 8e). In contrast, the yeast growth form was significantly more abundant in EFNs compared to FNs (*p* < 0.05).

For *H. stigonocarpa*, fungal communities associated with EFNs and the adjacent phyllosphere/endosphere comprised 11 primary lifestyles and 12 secondary lifestyles (Figure S6a,b). The plant pathogenic lifestyle was dominant in both plant microhabitats, while litter saprotrophs predominated at the secondary lifestyle level. No significant differences were detected between microhabitats for either primary or secondary lifestyles. Growth form patterns were similar across compartments, with dominance of filamentous fungi, followed by yeasts and, to a lesser extent, dimorphic yeasts (Figure S6c).

## 4 Discussion

Different plant compartments constitute distinct ecological niches for fungal communities, and the structural and physiological characteristics of each tissue play an important role in the taxonomic structure of these communities [68, 69, 70]. In this study, we investigated the fungal communities associated primarily with the floral and extrafloral nectaries of two woody plant species from the Brazilian Cerrado, *M. brasiliensis* and *H. stigonocarpa*. To our knowledge, this is one of the first studies to examine fungal communities associated with floral and extrafloral nectaries in this biome, and the first to apply an environmental DNA‐based approach to characterize the fungi inhabiting these plant microhabitats. Our results, therefore, provide new and comprehensive information on the diversity and taxonomic structure of fungal communities associated with nectaries, as well as the dominant fungal groups occupying these environments. In a previous study, we investigated the microbial communities associated with the extrafloral nectaries of *Calolisianthus speciosus* using scanning electron microscopy [28]. That work revealed complex microbial communities, including filamentous fungi, yeasts, and bacteria, occurring in greater abundance in the EFNs compared to adjacent plant surfaces, particularly the phyllosphere. The results presented here expand upon these observations, demonstrating that, for the plant species examined in this study, nectaries harbor fungal diversity that extends beyond the morphological diversity previously reported for *C. speciosus*. This discovery is particularly relevant because the microorganisms that inhabit nectaries have the potential to influence nectar quality [71], mediate ecological interactions, such as those involving pollinators and defensive mutualists, and affect population and community dynamics at multiple trophic levels [8, 11, 22, 26, 72]. Furthermore, the fungi that colonize nectaries are likely adapted to environments characterized by high sugar concentrations and strong osmotic stress [23], suggesting that these microorganisms may also represent promising candidates for biotechnological applications under osmotically challenging industrial conditions.

Sample‐level rarefaction analyses, together with rarefaction and extrapolation curves, indicate that the sampling effort employed for *M. brasiliensis* was sufficient to capture most of the fungal diversity associated with FNs and EFNs. The clear stabilization of the rarefaction curves and the limited gains predicted by extrapolation corroborate the robustness of diversity estimates and community‐level comparisons for this species [73, 74]. These results suggest that the observed patterns of taxonomic composition, diversity, and functional structure in *M. brasiliensis* are unlikely to be artifacts of undersampling, which lends confidence to the ecological inferences made for this host. In contrast, sampling completeness was lower for *H. stigonocarpa*, particularly for the phyllosphere/endosphere compartment. The rarefaction curves for this species did not consistently reach a plateau, indicating that part of the fungal diversity may remain unsampled. This limitation was further aggravated by the loss of one biological replicate from the phyllosphere/endosphere during sample transport, which reduced replication and statistical power for comparisons involving this compartment [55, 56, 75]. To mitigate the effects of uneven sequencing depth and incomplete sampling, we applied rarefaction [55, 56]. We emphasize that the characterization of the fungal community associated with *H. stigonocarpa* should be interpreted with caution.

In our hypotheses, we postulated that fungal communities associated with FNs and EFNs of *M. brasiliensis* would differ in taxonomic structure. This hypothesis was partially corroborated by the results obtained. At the phylum level, and for both plant species and microhabitats analyzed, Ascomycota was dominant. This pattern is expected, considering that this phylum includes the largest number of fungal species described to date [76, 77, 78] and that most of its representatives occur associated with plants, occupying different tissues and surfaces [79, 80].

The most evident differences between microhabitats were observed at the class level, both for the floral and extrafloral nectaries of *M. brasiliensis* and for the extrafloral nectaries and the phyllosphere/endosphere of *H. stigonocarpa*. In both systems, cosmopolitan genera, such as *Cladosporium*, were dominant across the different microhabitats analyzed. This result can be explained by the wide distribution of this genus, its high dispersal capacity, and its ability to colonize a broad range of substrates and environmental conditions [81]. Furthermore, *Cladosporium* species are frequently associated with insects [82], which constitute the main visitors to the nectaries evaluated in this study. Previous studies also report *Cladosporium* as one of the dominant genera in floral and extrafloral nectaries, as well as in fungal communities associated with insects [83, 84, 85, 86]. There is evidence that species of this genus, when colonizing nectar, can produce volatile organic compounds capable of positively influencing insect attraction [86]. In the FNs of *M. brasiliensis*, in addition to *Cladosporium*, other genera showed high relative abundance, such as *Lecanicillium* and *Erysiphe*. This pattern is partially consistent with our initial hypothesis, since the EFNs were dominated by *Cladosporium*. In contrast, in the microhabitats of *H. stigonocarpa*, the same taxonomic groups were dominant in both the EFNs and the phyllosphere/endosphere. This result may be related to the physical proximity between these structures and the high number of nectaries per leaf (Figure 2h‐j). Additionally, the nectaries of this species showed intense secretory activity and high visitation by ants (Figure 2b), which may have favored the dispersal of nectar onto the adjacent leaf surface, contributing to the homogenization of fungal composition among microhabitats.

The core mycobiota of the plant compartments analyzed in both species was characterized not only by the presence of *Cladosporium*, but also by other filamentous fungi commonly associated with Cerrado plants, such as *Didymella, Epicoccum*, and *Alternaria*. These taxa comprised the microbial core of both the nectaries and the phyllosphere/endosphere and are widely reported as endophytic fungi in plant species from this biome [47, 87]. A plausible explanation for this pattern is that, during environmental DNA extraction, some of these microorganisms colonize tissues adjacent to the nectaries, occurring as endophytes near or within the secretory region. It is also worth noting that representatives of these groups have been reported as components of the mycobiota associated with floral honeys and honeydew [84]. This overlap between nectariferous environments and nectar‐derived products raises new questions about the role of these fungi in nectar quality, as well as their potential influence on the physicochemical properties of this resource and, consequently, on the attraction or deterrence of pollinators. In addition to filamentous fungi, we observed the recurrent presence of yeast‐like genera, such as *Aureobasidium*, *Hannaella*, and *Starmerella*, composing the core of the mycobiota of nectaries and the phyllosphere/endosphere in both plant species analyzed. Yeast‐like fungi are frequently associated with these compartments, as nectar and adjacent surfaces constitute habitats rich in sugars and other easily assimilable compounds [88, 89, 90, 91]. Furthermore, some of these genera have been reported as endophytes of fruits from native Cerrado species [92, 93], suggesting that yeasts initially associated with nectar may, during plant development, colonize internal tissues and establish themselves as endophytes.

As part of our initial hypothesis, we expected the FNs of *M. brasiliensis* to harbor higher fungal richness and diversity than the EFNs, based on previous studies reporting that inflorescences are visited by multiple groups of pollinators, whereas EFNs are predominantly exploited by ants [94, 95, 96, 97, 98, 99]. However, it is important to note that pollinator and ant visitation were not directly quantified in the present study. Our results revealed an opposite pattern. EFN‐associated communities showed higher species richness and a composition significantly different from that observed in FNs. Moreover, despite the presence of a dominant group, EFNs exhibited higher evenness values, indicating a more uniform distribution of species. Although EFNs are mainly associated with ant visitation according to the literature, it is essential to consider that ant foraging behavior often occurs in soil and leaf litter [100, 101], environments known to harbor high fungal density and diversity [102, 103, 104]. During their movement across different microenvironments, ants may act as vectors for fungal spores, facilitating dispersal and potentially influencing the structure of fungal communities associated with EFNs [105, 106]. This mechanism may partially explain the higher richness and evenness observed in EFNs. Across the plant microhabitats of *H. stigonocarpa*, EFNs showed higher observed richness, whereas the phyllosphere/endosphere exhibited higher diversity values according to the Shannon, Simpson, and Pielou indices. These compartments are widely recognized as diverse microbial habitats [29, 107, 108]. It is important to note that the smaller sample size and incomplete sampling of phyllosphere communities, as indicated by rarefaction and extrapolation curves, may have limited the detection of diversity patterns across these compartments.

Beyond the differences observed in alpha diversity metrics between FNs and EFNs of *M. brasiliensis*, our results show that these microhabitats harbor distinct fungal communities. Beta diversity analyses, based on both abundance and presence–absence of fungal ASVs, together with beta diversity partitioning and indicator/specialist species indices, suggest that these compartments impose their own selective filters on fungal colonization. Although both Bray–Curtis and Jaccard analyses supported differentiation between floral and extrafloral nectaries, the significant heterogeneity in dispersion detected for Jaccard distances indicates that presence–absence patterns may also reflect variation in the distribution of rare taxa among samples, whereas abundance‐based differences were not influenced by dispersion effects. These patterns suggest that nectary‐associated microbial communities are not solely shaped by passive dispersal, but instead reflect selective processes linked to intrinsic features of these microhabitats. Among the factors potentially driving this selection are differences in nectar chemistry, visitor frequency and identity, and the temporal dynamics of nectar secretion. Previous studies have shown that floral and extrafloral nectar from the same plant species can differ markedly in their physicochemical and enzymatic properties [4, 94, 109, 110]. Visitor frequency also plays a central role, as these organisms act as the main vectors introducing microorganisms into nectaries [111, 112]. In addition, nectary structural traits may directly influence the composition of associated microbial communities [112, 113]. Other factors, such as seasonality and environmental variation, can further modulate these communities over time, acting as environmental filters on nectary‐associated microorganisms [5, 114]. For the host *H. stigonocarpa*, beta diversity analyses should be interpreted with caution, as the low sample size limits robust ecological comparisons.

Functional analysis indicated that the different plant compartments of both host species were dominated by fungi classified as plant pathogens and saprotrophs. Nevertheless, these results should be interpreted with caution, as functional inferences based solely on taxonomic assignments do not necessarily reflect actual pathogenic activity. In *M. brasiliensis*, floral nectaries showed a higher relative abundance of fungi classified as foliar endophytes and pathogens. This pattern may be associated with their close proximity to reproductive tissues, as well as with the possibility of colonization by endophytes and/or pathogens in these tissues [87, 115], which were also sampled in this study. In contrast, the greater representation of epiphytic fungi in extrafloral nectaries of this species may reflect the influence of phyllosphere communities, given that these microhabitats are located on the leaves of the host plant. Since the phyllosphere is typically dominated by fungi with epiphytic life strategies [116, 117], it is likely that these communities were partially captured during EFN sampling, thereby shaping the observed functional profile. We should also consider that the adjacent tissue sampled together with the NFEs may harbor endophytic fungi and also pathogens, which may have influenced our results.

Regarding growth form, we initially expected a predominance of yeasts in nectaries, as this fungal group has traditionally been described as a major component of nectar associated communities [4, 23, 88, 91, 118]. This expectation is largely based on the fact that nectaries represent sugar rich microhabitats, a condition commonly associated with the occurrence and success of yeast‐like fungi [21, 119]. However, across all plant microhabitats analyzed, fungal communities were consistently dominated by filamentous fungi. Although this pattern was not anticipated, filamentous fungi are also known to occur in nectariferous environments, even though their presence and ecological roles remain less explored when compared to yeasts [83, 84, 85, 86]. In addition, as nectaries were sampled together with portions of adjacent tissues, it is likely that foliar endophytes and epiphytic fungi, which are predominantly filamentous, were partially included in the samples, contributing to the observed dominance of this growth form. Therefore, these results may partially reflect the fungal communities associated with adjacent plant tissues rather than exclusively the ecological composition of fungi inhabiting the nectary environment. Significant differences between growth forms were detected only between extrafloral and floral nectaries of *M. brasiliensis*. In this case, filamentous fungi showed significantly different abundances between the two nectary types, whereas yeasts were more abundant in extrafloral nectaries compared to floral ones. This pattern may be linked to specific features of extrafloral nectar, whose composition and secretion dynamics may favor the establishment of yeast‐like fungi in these microhabitats.

Finally, our results demonstrate that floral and extrafloral nectaries can act as relevant microhabitats for fungal communities associated with the studied Cerrado woody plant, harboring diverse and structured communities shaped by both dispersal and local selective processes. Differences in diversity and composition between nectary types indicate that these microhabitats may contribute to structuring associated fungal communities through environmental filtering. Given the limitations of our sampling design and the lack of direct measurements of nectar composition and secretion dynamics, the mechanisms underlying these patterns remain to be further investigated. Our findings contribute to expanding current knowledge of fungal diversity associated with nectar‐related plant microhabitats and highlight the importance of considering multiple plant compartments when investigating plant–microbe interactions in biodiversity‐rich ecosystems such as the Brazilian Cerrado.

## Author Contributions

**Jefferson Brendon Almeida dos Reis:** conceptualization, funding acquisition, investigation, writing – original draft, writing – review and editing, visualization, validation, methodology, software, formal analysis, project administration, resources, supervision, data curation. **Sofia Coradini Schirmer:** conceptualization, investigation, funding acquisition, visualization, validation, methodology, writing – review and editing, formal analysis, software, data curation, supervision. **Helson Mario Martins do Vale:** conceptualization, investigation, funding acquisition, writing – review and editing, visualization, validation, project administration, resources, supervision, data curation.

## Conflicts of Interest

The authors declare no conflicts of interest.

## Supporting information

Supporting File

## Data Availability

The data that support the findings of this study are available from the corresponding author upon reasonable request.
