# Supplementary material for: Fungal Community Composition Varies Across Floral and Extrafloral Nectaries
Source: J Basic Microbiol. 2026 Aug 5;66(8):e70193. doi: 10.1002/jobm.70193 (PMC13439489; doi:10.1002/jobm.70193)
Supplement: Supplementary file 1 [file JOBM-66-e70193-s001.docx]

**Fungal community composition varies across floral and extrafloral nectaries**

Jefferson Brendon Almeida dos Reis1; Sofia Coradini Schirmer2; Helson Mario Martins do Vale3

1 Postgraduate Program in Microbiology Biology, Department of Phytopathology, Institute of Biological Sciences, University of Brasília (UnB), Brasília, DF, Brazil

2 Postgraduate Program in Ecology, Department of Zoology, Institute of Biological Sciences, University of Brasília (UnB), Brasília, DF, Brazil

3Department of Phytopathology, Institute of Biological Sciences, University of Brasília (UnB), Brasília, DF, Brazil

*Corresponding author

1 Email: jeffersonalmeidareis@gmail.com

1 orcid: <https://orcid.org/0000-0002-2768-214X>

**Supplementary Table 1. Summary of sequencing data quality metrics as reported by the sequencing service provider**

| **Sample** | **Group** | **Plant** | **RawPE** | **Combined** | **Qualified** | **Nochime** | **Base (nt)** | **Avglen (nt)** | **GC** | **Q20** | **Q30** |
| --- | --- | --- | --- | --- | --- | --- | --- | --- | --- | --- | --- |
| JF1 | Phylosphere/Endosphere | *Hymenaea stigonocarpa* | 215859 | 152870 | 149181 | 125863 | 39217430 | 311.59 | 63.26% | 96.83% | 91.91% |
| JF2 | Phylosphere/Endosphere | *Hymenaea stigonocarpa* | 202968 | 137328 | 134536 | 102563 | 32524223 | 317.11 | 64.96% | 97.68% | 92.77% |
| JN1 | Extrafloral nectary | *Hymenaea stigonocarpa* | 204598 | 157350 | 152251 | 135520 | 40516187 | 298.97 | 61.20% | 97.23% | 92.68% |
| JN2 | Extrafloral nectary | *Hymenaea stigonocarpa* | 206261 | 163161 | 158305 | 136473 | 40379719 | 295.88 | 61.21% | 97.27% | 92.82% |
| JN3 | Extrafloral nectary | *Hymenaea stigonocarpa* | 308309 | 273602 | 266991 | 239334 | 68635315 | 286.78 | 60.65% | 96.88% | 92.11% |
| NEF1 | Extrafloral nectary | *Maprounea brasiliensis* | 152716 | 148225 | 147284 | 145772 | 39539555 | 271.24 | 52.14% | 99.38% | 97.56% |
| NEF2 | Extrafloral nectary | *Maprounea brasiliensis* | 174626 | 172115 | 170674 | 168461 | 47577993 | 282.43 | 53.01% | 99.30% | 97.07% |
| NEF3 | Extrafloral nectary | *Maprounea brasiliensis* | 206465 | 201644 | 200208 | 196137 | 52991114 | 270.17 | 52.21% | 99.30% | 97.13% |
| NEF4 | Extrafloral nectary | *Maprounea brasiliensis* | 204610 | 202705 | 200828 | 196458 | 59143886 | 301.05 | 54.16% | 99.26% | 97.17% |
| NEF5 | Extrafloral nectary | *Maprounea brasiliensis* | 204261 | 194131 | 193164 | 189601 | 50672024 | 267.26 | 52.06% | 99.38% | 97.33% |
| NF1 | Floral nectary | *Maprounea brasiliensis* | 131085 | 127773 | 127353 | 125408 | 31076204 | 247.80 | 50.42% | 99.57% | 98.07% |
| NF2 | Floral nectary | *Maprounea brasiliensis* | 202053 | 197348 | 196411 | 194339 | 52367711 | 269.47 | 52.48% | 99.48% | 97.89% |
| NF3 | Floral nectary | *Maprounea brasiliensis* | 203982 | 198166 | 197971 | 194227 | 44982454 | 231.60 | 48.72% | 99.71% | 98.58% |
| NF4 | Floral nectary | *Maprounea brasiliensis* | 206408 | 204778 | 203282 | 202578 | 56220459 | 277.52 | 53.55% | 99.31% | 97.29% |
| NF5 | Floral nectary | *Maprounea brasiliensis* | 128163 | 124244 | 123682 | 122235 | 32006247 | 261.84 | 51.89% | 99.48% | 97.83% |

**Supplementary Table S1. Summary of sequence processing and quality filtering steps performed using the DADA2 pipeline**

| **Sample** | **Group** | **Plant** | **Input** | **Filtered** | **Denoised Forward** | **Denoised Reverse** | **Merged** | **Nonchim** |
| --- | --- | --- | --- | --- | --- | --- | --- | --- |
| JF1 | Phylosphere/Endosphere | *Hymenaea stigonocarpa* | 215851 | 146309 | 146123 | 146080 | 133808 | 131252 |
| JF2 | Phylosphere/Endosphere | *Hymenaea stigonocarpa* | 202956 | 121352 | 121085 | 121120 | 113317 | 102203 |
| JN1 | Extrafloral nectary | *Hymenaea stigonocarpa* | 204595 | 133390 | 133166 | 133131 | 129223 | 125461 |
| JN2 | Extrafloral nectary | *Hymenaea stigonocarpa* | 206259 | 139471 | 139142 | 139268 | 134914 | 130740 |
| JN3 | Extrafloral nectary | *Hymenaea stigonocarpa* | 308113 | 263770 | 263398 | 263379 | 237037 | 229041 |
| NEF1 | Extrafloral nectary | *Maprounea brasiliensis* | 152473 | 143386 | 142985 | 142663 | 140741 | 137639 |
| NEF2 | Extrafloral nectary | *Maprounea brasiliensis* | 174318 | 161028 | 160448 | 160405 | 157574 | 156448 |
| NEF3 | Extrafloral nectary | *Maprounea brasiliensis* | 206084 | 191055 | 190127 | 190155 | 187010 | 183616 |
| NEF4 | Extrafloral nectary | *Maprounea brasiliensis* | 204255 | 193144 | 192556 | 192429 | 189452 | 187376 |
| NEF5 | Extrafloral nectary | *Maprounea brasiliensis* | 203910 | 183608 | 183151 | 182675 | 181010 | 179318 |
| NF1 | Floral nectary | *Maprounea brasiliensis* | 130886 | 120575 | 120175 | 120330 | 119032 | 117027 |
| NF2 | Floral nectary | *Maprounea brasiliensis* | 201745 | 189370 | 188916 | 188859 | 186783 | 180210 |
| NF3 | Floral nectary | *Maprounea brasiliensis* | 203683 | 187477 | 187094 | 186942 | 184475 | 170609 |
| NF4 | Floral nectary | *Maprounea brasiliensis* | 206069 | 193577 | 193435 | 193463 | 192384 | 191668 |
| NF5 | Floral nectary | *Maprounea brasiliensis* | 127916 | 118456 | 118216 | 118065 | 116843 | 112348 |

**Supplementary Figure 1 Rarefaction curves illustrating sequencing depth for fungal communities associated with different plant compartments and microhabitats of *Maprounea brasiliensis* and *Hymenaea stigonocarpa*.** a) Rarefaction curves for floral and extrafloral nectaries of *M. brasiliensis*; b) phyllosphere/endosphere and extrafloral nectaries of *H. stigonocarpa*. The rarefaction curves indicate that sequencing depth for fungal communities associated with the different plant compartments of *M. brasiliensis* reached a plateau, suggesting sufficient sequencing coverage across all samples. In contrast, for *H. stigonocarpa*, not all samples reached a plateau, indicating that the sequencing effort was insufficient to fully capture fungal diversity in some compartments. NF: floral nectaries; NFE: extrafloral nectaries; JF: phyllosphere/endosphere; JN: extrafloral nectaries

**Supplementary Figure 2 Rarefaction and extrapolation curves showing sequencing depth and estimated fungal richness (q0= richness; ASVs: Amplicon Sequence Variants) for communities associated with different plant compartments and microhabitats.** a) Floral and extrafloral nectaries of *Maprounea brasiliensis*. b) Extrafloral nectaries and the adjacent phyllosphere/endosphere of *Hymenaea stigonocarpa*. Solid lines represent rarefaction based on observed sequencing effort, whereas dashed lines indicate extrapolation beyond the sampled depth. The stabilization of the curves indicates sufficient sequencing coverage, while non-asymptotic trends suggest incomplete sampling of fungal diversity in some compartments.

**Supplementary Figure 3 Alpha diversity metrics of fungal communities associated with different microhabitats of two plant species from the Brazilian Cerrado.** a) Observed richness (Observed), Pielou's evenness, and Shannon and Simpson indices for communities associated with extrafloral nectaries (EFNs) and floral nectaries (FNs) of *M. brasiliensis*. b) Observed richness (Observed), Pielou's evenness, and Shannon and Simpson indices for communities associated with the phyllosphere/endosphere (Phyllos/Endos) and EFNs of *H. stigonocarp*a. Asterisks indicate statistically significant differences between microhabitats (Wilcoxon test, adjusted p < 0.05).

**Supplementary Figure 4** **Partitioning of Sørensen beta diversity among the analyzed plant compartments.** a) *Maprounea brasiliensis*: total beta diversity (βSOR), species turnover (βSIM), and nestedness (βSNE) between fungal communities associated with floral nectaries (FNs) and extrafloral nectaries (EFNs). b) *Hymenaea stigonocarpa*: total beta diversity (βSOR), turnover (βSIM), and nestedness (βSNE) among fungal communities associated with extrafloral nectaries (EFNs), the phyllosphere/endosphere (Phyllos/Endos). Values represent pairwise comparisons between samples, and color intensity indicates the degree of compositional dissimilarity, according to the scale shown.

**Supplementary Figure 5 Core structure of the mycobiome associated with different plant microhabitats of *Maprounea brasiliensis* and *Hymenaea stigonocarpa*.** Panels a) and b) show changes in core size (number of taxa) as a function of increasing relative abundance detection thresholds, based on prevalence criteria (70%), for *M. brasiliensis* and *H. stigonocarpa*, respectively. Panels c) and d) show genus-level prevalence heat maps across detection thresholds for *M. brasiliensis* and *H. stigonocarpa*, respectively.

**Supplementary Figure 6 Functional profiles of fungal communities associated with extrafloral nectaries (EFNs) and adjacent phyllosphere/endosphere (Phyllos/Endos) of *Hymenaea stigonocarpa*.** a) Heatmap and hierarchical clustering of primary lifestyles based on relative abundance (%). b) Heatmap and hierarchical clustering of secondary lifestyles based on relative abundance (%). c) Relative abundance (%) of fungal growth forms (filamentous mycelium, yeast, and dimorphic yeast) across samples from Phyllos/Endos and EFNs.
